# The effect of high‐polyphenol sumac (*Rhus coriaria*) on food intake using sensory and appetite analysis in younger and older adults: A randomized controlled trial

**DOI:** 10.1002/fsn3.3369

**Published:** 2023-04-18

**Authors:** Nasim Soleymani Majd, Shelly Coe, Helen Lightowler, Pariyarath Sangeetha Thondre

**Affiliations:** ^1^ Oxford Brookes Centre for Nutrition and Health (OxBCNH), Department of Sport, Health Sciences and Social Work, Faculty of Health and Life Sciences Oxford Brookes University Oxford UK

**Keywords:** antioxidant, food intake, older adults, polyphenol, *Rhus coriaria* sumac, sensory

## Abstract

Aging is accompanied by a decline in appetite and food intake with associated deficiencies in both macronutrients and micronutrients. The aim of this study was to investigate the impact of adding Iranian brown sumac *(Rhus coriaria)* (CIBS) into butternut squash soup on sensory evaluation and food intake among older adults (*n* = 20; >65 years old) and younger adults (*n* = 20; 18–35 years old). To evaluate the polyphenol content and antioxidant activity of the sumac samples, a Folin–Ciocalteu assay (FCR) and ferric ion reducing antioxidant power (FRAP) assay were used, respectively. L‐glutamic acid was assessed using a Megazyme L‐glutamic acid assay. Compusense software was used to assess the sensory evaluation attributes of free‐living older adults and younger adults receiving different doses of sumac in butternut squash soup. Nutritics software was used to assess food intake following the addition of 0.37 g of sumac to soup. CIBS was selected based on a preliminary assessment in vitro for L‐glutamic acid, antioxidant, and polyphenol content of six varieties of sumac. Sensory evaluation results revealed that the difference in perceived intensity of brown color between the soup samples with different doses of CIBS was greater in the younger adults' group (*p* = .001) than in older adults (*p* = .037). In addition, the food intake study found that during the ad libitum lunch, older adults consumed more energy (kcal; *p* = .014), protein (g; *p* = .025), carbohydrate (g; *p* = .013), and fat (g; *p* = .002) after soup with sumac compared to control soup. The overall findings of this study suggest that the addition of sumac to food may have a potential benefit in enhancing ad libitum lunch intake in older adults leading to effective management of malnutrition. This may promote healthy aging and minimize the burden and the consequences of anorexia of aging as main public health concerns.

## Graphical abstract

This article investigated the impact of adding polyphenol and glutamic acid‐rich Iranian brown sumac into butternut squash soup on sensory attributes and food intake among older and younger adults. The results showed that soup with sumac improved energy and macronutrient intake in older adults at an ad libitum lunch compared to a control soup. The overall findings of this study suggest that the addition of sumac to food may have a potential benefit in enhancing ad libitum lunch intake in older adults leading to effective management of malnutrition.

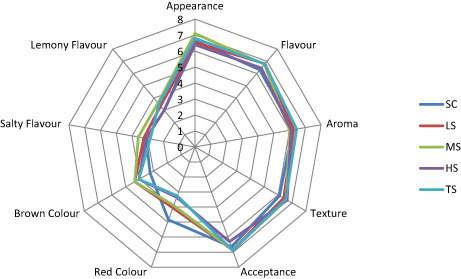

## 1 INTRODUCTION

Herbs and spices were traditionally used to enhance the taste of food, as well as for medicinal purposes (Kaefer & Milner, 2008). They can also be added into low‐salt dishes to improve appetite and food intake (Dougkas et al., 2019). Many traditional dishes are well known for their signature herbs and spices that enrich the taste and aroma of food and offer health benefits due to the presence of bioactive phenolic compounds (Asgharpanah & Saatti, 2014; Egharevba & Gamaniel, 2017).

Epidemiological studies have confirmed the association between a high level of antioxidant consumption and decreased rates of mortality and morbidity (Devasagayam et al., 2004). As a result, the enhancement of foods using natural antioxidants and polyphenols has been acknowledged by food researchers (Bashash et al., 2012). Herbs and spices contain significant concentrations of free glutamic acids (Aremu et al., 2011). It has been found that nonessential amino acids (including glutamates) play a role as umami substances to enhance the taste of food by activating the chemical‐detecting components of taste buds (Mouritsen, 2012).

Sumac *(Rhus coriaria)* is a spice that grows in the wild tropical regions including Mediterranean coastlines from the Canary Islands to Iran (Abu‐Reidah et al., 2015; Rayne & Mazza, 2007) and it is commonly used in Middle Eastern dishes for seasoning and flavoring (Zargham & Zargham, 2008). Although sumac has some health benefits including antimicrobial (Fazeli et al., 2007; Sagdic & Ozcan, 2003) and antioxidant activity (Bozan et al., 2003; Kosar et al., 2007), antihyperlipidemic activity and hypoglycemic effects (Anwer et al., 2013; Madihi et al., 2013; Shafiei et al., 2011) uncertainty remains about the relationship between the consumption of sumac, appetite, and food intake. It is pertinent to investigate this in the context of aging when threshold perceptions of taste and flavor of food are altered resulting in malnutrition among older adults (Methven et al., 2012). The benefits of using flavoring agents, including herbs and spices, to reduce salt intake resulting in a reduction in cardiovascular diseases, are noteworthy in healthy aging (Anderson et al., 2015).

The literature emphasizes the importance of older adults consuming food with sufficient energy in order to maintain their immune system levels and muscle mass (Baum et al., 2016). An increase in daily protein intake for healthy adults is recommended to almost 1.0 g/kg of body weight, and for those who are either malnourished or at risk of malnutrition, the amount should increase by half a gram/kg (Deutz et al., 2014). Previous research has investigated methods that help to increase food consumption in the older population, with some studies demonstrating the impact of added herbs and spices on improving the liking and palatability of food (Dermiki et al., 2015; Dougkas et al., 2019; Fritts et al., 2018; Peters et al., 2014). Moreover, monosodium glutamate (MSG) has been added to different foods, resulting in flavor enhancement and increased appetite (Dermiki et al., 2013; Dermiki et al., 2015; Schiffman, 2000). Hence, it can be hypothesized that sumac, as a potential natural flavor enhancer containing glutamic acid, might also increase appetite among older adults. The aim of this research was to identify sumac with high polyphenol and glutamic acid content for use as a food flavoring to enhance food intake in older adults and compare the results with younger adults.

## 2 METHODS

### 2.1 Materials

#### 2.1.1 In vitro studies

All chemicals and reagents were purchased from Sigma Aldrich and the Megazyme Co. Commercial Turkish sumac (CTS; Whole Foods Online Ltd), Commercial Palestinian sumac (CPS; YAFFA Ltd), Commercial Iranian red sumac (CIRS; Mahan products), Commercial Iranian brown sumac (CIBS; Donya Company), Fresh red sumac (FRS; London), and Fresh brown sumac (FBS; Kordestan‐Iran) were used in this study. All sumac samples belonged to the *Rhus coriaria* species.

#### 2.1.2 In vivo studies

The test food used was butternut squash soup prepared using 25% reduced salt vegetable stock cubes. Pasta, vegetable oil, and tomato sauce were used for the ad libitum lunch. All these ingredients were purchased from local supermarkets.

### 2.2 In vitro testing of sumac samples for antioxidants, polyphenols, and L‐glutamic acid

The FRS and FBS were first air‐dried for 4 weeks, away from direct light and then ground into powder using mortar and pestle. The extraction for polyphenols was completed by adding 0.2 g of sumac sample into 4 mL of the solvents–distilled water, 80% acetone, and 80% ethanol. The samples were transferred to a shaking water bath and then incubated for 2 h at room temperature away from light. The samples were then centrifuged at 3000 rpm for 10 min (Heraeus Instruments, D‐37520 Osterode) to separate the supernatant used as sumac extract. The total antioxidant capacity (TAC) of each sumac extract was assessed using a Ferric ion reducing antioxidant power (FRAP) assay (Benzie & Strain, 1996; Ryan et al., 2011). The total phenolic content (TPC) of each sumac extract was analyzed using the method described by Coe et al. (2013). L‐glutamic acid was measured using L‐glutamic acid Megazyme assay procedure (K‐GLUT 11/15) following the extraction of 0.2 g of sumac samples in 4 mL of distilled water. The results for L‐glutamic acid in sumac samples were calculated as g/100 g (Aremu et al., 2011; Sakhr & El Khatib, 2020). The average g glutamic acid/100 g protein of each sample was calculated, as shown below:

$$\frac{\text{average of absorbance}\times 6.75}{100}=\left(g/g\;\text{protein}\right).$$

$$\left(g/g\;\text{protein}\right)\times 100=g\;\text{glutamic acid}/100\;g\;\text{protein}.$$

### 2.3 In vivo testing

#### 2.3.1 Sensory evaluations of different doses of sumac

The test foods comprised a control soup (SC) without sumac and test soups with four different doses of CIBS sumac: 0.25% (low‐dose soup; LS), 0.50% (medium‐dose soup; MS), 0.75% (high‐dose soup; HS), and 1% (total dose soup; TS). The soup was prepared with butternut squash (500 ± 10 g), low‐salt vegetable stock (4.5 ± 0.2 g), and water (1 L). Each participant received 100 mL of soup in a 4 oz polystyrene pot container. The doses of CIBS sumac were added to the containers before the warm soups were poured into containers and mixed well with the soup by stirring, ahead of serving. Participants were blinded to the samples that were served in both sessions with at least a 2‐day wash‐out in between; three samples in the first and two in the second. The samples were coded using the Compusense software (Compusense Inc), and served at room temperature along with water and three crackers for cleansing participants' palates between the soup samples. All sessions were carried out at room temperature (21°C) in individual sensory booths lit by artificial light at Oxford Brookes Centre for Nutrition and Health (OxBCNH).

#### 2.3.2 Food intake study

Participants were given 150 g of butternut squash soup in each session: with no added sumac (SC), 1% CIBS added at the end of cooking (SSE), or 1% CIBS added during cooking (SSC). The soup was heated for 30 min before serving. The participants were provided with an ad libitum lunch (pasta with tomato sauce), 10 min after the soup was served. The participants were blinded to the soup sample they received. The pasta was weighed (180 ± 1 g) and served along with a glass of water (340 mL) at room temperature (Peters et al., 2014). Participants were instructed to eat as much pasta and drink as much water as they desired.

### 2.4 Participants

A study poster was used to recruit participants from different venues. All participants were asked to attend the OxBCNH for three test sessions. Volunteers received a participant information sheet (PIS) via email, mail, or in person. A signed consent form was returned prior to the first test session. All participants completed a health questionnaire; older adult participants were also asked to complete a Functional Ability Health Questionnaire (Hall et al., 2011). Weight (kg) and height (cm) were self‐reported by the participants. They were instructed not to eat or drink (except water) for 1 h before the test commenced.

### 2.5 Protocols

#### 2.5.1 Sensory evaluation

Ethical approval for the current study was granted by the University Research Ethics Committee (UREC) of Oxford Brookes University (Registration No: 161059). A total of 40 participants were recruited, between July 2017 and December 2017, based on the following inclusion criteria (Older adults aged >65 years, younger adults aged 18–35 years old, no allergies to herbs, spices, or any types of vegetables, nonsmoker, no cold or hay fever on test day, no diseases or medication which affects sensory perception, ability to read and understand English, and ability to attend, stand, and sit for up to an hour) and separated into two groups. The older adults group had 20 free‐living participants (11 females and 9 males) aged over 65 years and the younger adult group comprised 20 participants (13 females and 7 males) aged 18–35 years old. The study was a randomized, crossover repeated measures design using a 9‐point hedonic scale method for measuring degrees of liking, whereby numerical values were assigned for verbal classification (Wichchukit & O'Mahony, 2015). A second set of questions validated their perceptions of liking and disliking each sample using open‐text comments. The hedonic test was designed using the Compusense software (Compusense Inc).

#### 2.5.2 Food intake

Ethical approval for this study was granted by the University Research Ethics Committee (UREC) of Oxford Brookes University on 13 March 2018 (Registration No: 181174). The study was retrospectively registered with Clinical Trials.Gov (NCT05534152). A total of 40 healthy male and female participants were recruited, between July 2018 and March 2020, based on the following inclusion criteria (Older adults aged >65 years, younger adults, aged 18–35 years old, no allergies to herbs, spices, or any types of vegetables, nonsmoker, no cold or hay fever on test day, no diseases or medication which affects sensory peception, ability to read and understand English, and ability to attend, stand, and sit for up to an hour) through several avenues and divided into two groups. Each group had 20 participants. In the older adults group, who were free living, 11 healthy females and nine healthy males took part in the study; in the younger adults group, 15 females and five males participated. The energy and macronutrient intake at the baseline were calculated based on 24‐h dietary recall by the participants. In this randomized, crossover repeated measures design study, the participants were randomly allocated to different sessions using Research Randomizer (Urbaniak & Plous, 2013) and recommended a breakfast (86–247 kcal) for the test day. Participants were advised to consume the same quantity of their preferred breakfast prior to each test session. In the first test session, participants were asked to complete a 24‐h dietary intake record to determine baseline energy and nutrient intake. To calculate energy and macronutrient intake from completed dietary records, Nutritics software version 5.022 (Nutritics Ltd) was used.

Soup samples were compared based on different methods for the addition of CIBS. The energy and macronutrient profile of the test soups with sumac and the ad libitum lunch served in each test session are given in Table 1.

**TABLE 1:** Nutritional composition of butternut squash soups with 1% CIBS per portion and the pasta served for ad libitum lunch.

Nutrient	Pasta (180 g)	Soup (150 g)	
		SC	SSE/SSC
Energy (kcal)	227	56	58
Carbohydrate (g)	37.8	12.1	12.3
Sugar (g)	4.2	6.6	6.6
Protein (g)	6.1	1.6	1.7
Fat (g)	5.7	0.2	0.2
Saturated fat (g)	0.5	0	0

Abbreviations: SC, control soup; SSC, sumac added during cooking; SSE, sumac added at the end.Data were calculated using Nutritics software.

### 2.6 Statistical analysis and sample size

Data were recorded in Microsoft Excel 2010 and statistical analysis was conducted using the Statistical Package for the Social Sciences (SPSS; version 23 and 25 USA). Data were analyzed for normality using Shapiro–Wilk's tests. The results are presented as mean ± standard deviation (SD). The significance level was set to *p* < .05.

#### 2.6.1 In vitro studies

Based on the results for normally distributed data, a parametric one‐way analysis of variance (ANOVA) with a post hoc test (using Tukey adjustment) was applied to determine significant differences within sumac samples and between solvents. The significance level was set to *p* < .05. For data not normally distributed, the results were analyzed using a nonparametric Kruskal–Wallis test between sumac samples' types, and the significance level was set to *p* < .05. In order to assess the relationship between the antioxidant activity and polyphenol content of all the sumac samples, a Pearson correlation test was run.

#### 2.6.2 In vivo sensory evaluation

A nonparametric Friedman test was used to evaluate the differences in sensory attributes between soup samples in older and younger adult groups, while a Wilcoxon test was run to determine differences in pairwise comparisons. Comparisons between the average intake of energy and macronutrients for older and younger adults were done using a Mann–Whitney test. The sample size determination was conducted using G*power (Version 3.1.9.7) which indicated that for a medium effect size of *d* = 0.5, α of 0.05 and power of (1‐ß) 0.80, a total of 35 participants was required.

#### 2.6.3 In vivo food intake

To analyze the impact of adding CIBS (SSE and SSC) to energy and nutrient intakes within older and younger adult groups, repeated measures ANOVA was applied for normally distributed data. Furthermore, comparisons of food intake between the two groups of adults were done using a Mann–Whitney test, or an independent t‐test depending on the normality of the data. In this study, G*power (v. 3.1.9.7) indicated that for a two‐tailed test with a medium effect size of *d* = 0.5, α of 0.05 and a power of (1–ß) 0.95, a sample size of 16 was required in each group to detect a difference in energy intake of 45 kcal (Flood & Rolls, 2007).

## 3 RESULTS

### 3.1 In vitro testing of sumac samples for antioxidants, polyphenols, and L‐glutamic acid

Table 2 shows the mean (±SD) total antioxidant activity (mol/L) and polyphenol content (mg GAE/g) of each type and form of sumac (CTS, CPS, CIRS, CIBS, FRS, and FBS) extracted by three solvents (water, 80% acetone, and 80% ethanol). Significant differences were observed between the solvents and each sumac sample (*p* < .05) with the highest antioxidant activity for 80% ethanol. Moreover, compared with other commercial sumac samples, CIBS followed by FBS showed higher activities (*p* < .05) in all three assays. There was a significant (*p* < .01), strong positive correlation between the polyphenol content and antioxidant activity of the sumac samples in all solvents: water, *r* = 0.813; 80% acetone, *r* = 0.887; and 80% ethanol, *r* = 0.623.

**TABLE 2:** Total antioxidant activity (mol/L), polyphenol content (mg GAE/g), and L‐glutamic acid (g glutamic acid/100 g protein) of each type and form of sumac.

Sumac type	Assay	Water	80% acetone	80% ethanol
CTS^a^	Antioxidant	3.5** ± 0.2	10.4** ± 2.6	25.9** ± 7.8
	Polyphenol	1.4^****^ ± 0.4	1.7^****^ ± 0.1	0.5^****^ ± 0.3
	L‐glutamic acid	0.7 ± 0.1	‐	‐
CPS^b^	Antioxidant	0.8**±0.1	4.2^**a^ ± 0.3	12.9^**a^ ± 2.9
	Polyphenol	0.1^****a^ ± 0.1	1.7^****a^ ± 0.1	0.5^****a^ ± 0.3
	L‐glutamic acid	0.2 ± 0.1	‐	‐
CIRS^c^	Antioxidant	1.2** ± 0.4	4.3^**a^ ± 0.7	9.1^**a^ ± 3.5
	Polyphenol	0.4^****a^ ± 0.2	1.8^****a^ ± 1.8	0.4^****a^ ± 0.2
	L‐glutamic acid	0.5 ± 0.2	‐	‐
CIBS^d^	Antioxidant	9.1^**a,b,c^ ± 2.7	13.9^**a,b,c^ ± 2.1	27.6^**b,c*^ ± 6.3
	Polyphenol	1.5^****a,b,c^ ± 0.7	5.1^****a,b,c^ ± 4.8	2.7^****b,c^ ± 0.6
	L‐glutamic acid	0.8 ± 0.3	‐	‐
FRS^e^	Antioxidant	7.3^***a,b,c^ ± 3.1	10.8^***b,c,d^ ± 1.5	14.4^***d^ ± 5.9
	Polyphenol	1.01^****b,c,d^ ± 0.5	4.5^*****a,b,c^ ± 4.5	2.1^*****^ ± 0.8
	L‐glutamic acid	0.7 ± 0.2	‐	‐
FBS^f^	Antioxidant	14.1^**a,b,c,d,e*^ ± 4.9	14.2^**a,b,c,e*^ ± 2.1	27.4^**b,c,e^ ± 8.6
	Polyphenol	2.4^*****a,b,c,d,e*^ ± 1.3	5.4^*****a,b,c,d*^ ± 5.3	3.4^*****b,c*^ ± 1.4
	L‐glutamic acid	1.3* ± 0.2	‐	‐

Abbreviations: CIBS, commercial Iranian brown sumac; CIRS, commercial Iranian red sumac; CPS, commercial Palestinian sumac; CTS, commercial Turkish sumac; FBS, fresh brown sumac; FRS, fresh red sumac; GAE, gallic acid equivalent. Data are presented as mean ± SD of triplicate measurements. The superscripts ^(a,b,c,d,e,f)^ show sumac types that are significantly different at *p* < .05. *is significantly higher than the other samples (one‐way ANOVA test, at *p* < .05). **is significantly different between solvents at *p* < .001. ***is significantly different between solvents at *p* < .003. ****is significantly different between solvents (one‐way ANOVA at *p* < .05) (polyphenol). *****is significantly different between solvents (Kruskal–Wallis test, at *p* < .01) (polyphenol). Column values with no superscript are not significantly different (*p* > .05).

### 3.2 Demographic characteristics of the participants In In vivo studies

Forty participants (20 older adults and 20 younger adults) volunteered to take part in the sensory evaluation study (Table 3). The results revealed, understandably, a significant difference between the ages of both groups of adults (*t* (38) = −32.6, *p* = .001, *d* = 10.4). On average, the age of older adults, in the food intake study, was higher than that of younger adults, with a large effect size of *d* = 8.9 (*t* (38) = −26.8, *p* = .001). However, no difference was observed in the comparison between the weight and height of older and younger adults in both sensory evaluation and food intake studies (*p* > .05).

**TABLE 3:** Demographic characteristics of older adults and younger adults in sensory evaluation and food intake studies (mean ± SD).

Characteristics	Sensory evaluation			Food intake		
	Older adults mean ± SD	Younger adults mean ± SD	*p*‐value	Older adults mean ± SD	Younger adults mean ± SD	*p*‐value
Age (years)	71.3 ± 4.3	27.5 ± 4.2	**.001** ^**a**^	72.1 ± 5.6	26.4 ± 5.1	**.001** ^**a**^
Weight (kg)	69.9 ± 11.4	68.3 ± 10.9	.654^a^	72.5 ± 10.5	76.9 ± 33.1	.581^a^
Height (cm)	171 ± 14	165.3 ± 9.9	.155^b^	169.8 ± 9.5	156.7 ± 35.4	.123^a^

*Note*: Data are shown as mean ± SD and tested for normality using a Shapiro–Wilk's test, bold figures show statistically significant difference at *p* < .05. An independent *t*‐test^a^ or Mann–Whitney^b^ test analyzed the baseline characteristics between older and younger adults.

### 3.3 Sensory evaluation of soup samples with different doses of sumac: Hedonic test

#### 3.3.1 Older adults

**FIGURE 1:**
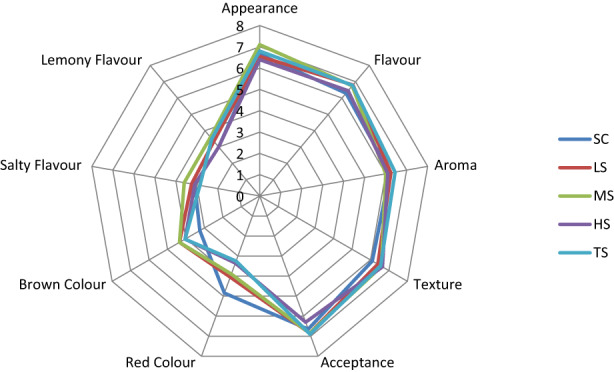
Spider chart for the sensory profile of butternut squash soup, with added CIBS, for older adults (over 65 years). The liking and intensity attributes were ranked from 1 to 9. SC: control soup; LS (0.25%): low‐dose sumac; MS (0.5%): medium‐dose sumac; HS (0.75%): high‐dose sumac; and TS (1%): total dose sumac.

The mean scores for the liking and intensity attributes of all the soup samples rated by older adults are presented in Figure 1. A Friedman test showed no significant differences (*p* > .05) between the soup samples for any of the liking attributes or the intensity of lemony flavor, salty flavor, or red color. However, the addition of sumac to soup samples resulted in an increased intensity of perceived brown color compared with the SC sample (*p* < .037); (χ^2^ (4) =10.2) among older adults. Despite an overall positive difference, a pairwise comparison showed no statistical differences between the soup samples (*p* > .05).

#### 3.3.2 Younger adults

**FIGURE 2:**
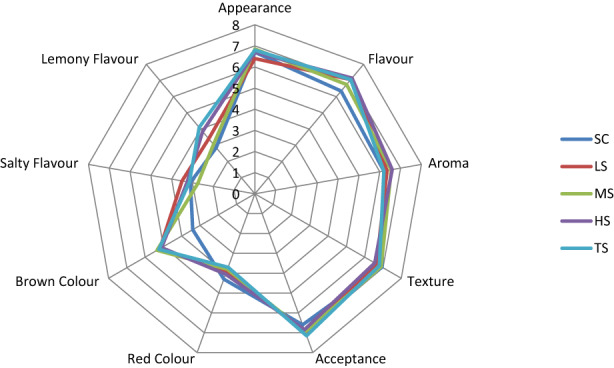
Spider chart for the sensory profile of butternut squash soup, with added CIBS, for younger adults (18–35 years old). Liking and intensity were ranked from 1 to 9. SC: control soup; LS (0.25%): low‐dose sumac; MS (0.5%): medium‐dose sumac; HS (0.75%): high‐dose sumac; and TS (1%): total dose sumac.

The mean scores for the liking attributes' (acceptance, texture, aroma, flavor, appearance) and intensity attributes' (lemony flavor, salty flavor, brown color, red color) for different soup samples in the younger adults group are presented in Figure 2.

A Friedman test identified a statistical difference in the intensity of the brown color of CIBS soup samples compared to SC soup among younger adults (*x* ^2^(4) = 23.5; *p* = .001; 2‐tailed). Pairwise comparisons revealed a large effect between the SC samples with LS and HS (*T* = −1.6, *r* = −.5; *p* = .012), SC with TS (*T* = −1.7, *r* = .5; *p* = .006), and SC with MS (*T* = −1.9, *r* = −0.6; *p* = .001).

Overall, analysis of the effect of different doses of sumac in soup samples compared with an SC sample showed no differences in liking and intensity of any attributes between older and younger adults, except for the brown color of soup TS, at *p* = .028, *t* (38) = 2.3, and *d* = 1.3.

### 3.4 Food intake following the soup samples with sumac

Unlike the younger adults, the older adults did not consume their soup fully in all sessions. Yet, there were no statistical differences observed in soup intake (*p* = .907) between the test sessions among older adults. The consumption of ad libitum pasta showed no statistical differences between the test sessions among older or younger adults, *p* = .075 and *p* = .49, respectively.

#### 3.4.1 Energy and nutritional profile of the ad libitum lunch, evening meal, and the all‐day food intake among older adults and younger adults

Results of ad libitum lunch among older adults showed a significant difference between the test sessions for energy (kcal, χ^2^(20) = 8.6; *p* = .014), protein (g, χ^2^(20) = 7.4; *p* = .025), carbohydrates (g, χ^2^(20) = 8.7; *p* = .013), and fat (g, χ^2^(20) = 12.6; *p* = .002) with higher intakes in the SSC session compared with SSE (Table 4). Comparison of the multiple tests showed that the SSE and SSC in comparison with the SC session had no impact on the intake of energy and macronutrients (*p* > .05). There was no impact on evening meal intake following the addition of CIBS sumac to soup samples between both groups of adults (*p* > .05). No statistical differences were observed in energy and macronutrient profiles of the all‐day food intake between the test sessions in either older adults or younger adults.

**TABLE 4:** Ad libitum lunch, evening meal, and the all‐day energy and macronutrient intake on three test sessions (SC, SSE, and SSC) within older adults and younger adults.

Nutrients		Ad libitum lunch				Evening meal				All‐day			
		SC	SSE	SSC	*p*‐value	SC	SSE	SSC	*p*‐value	SC	SSE	SSC	*p*‐value
Energy (kcal)	O	185.3 ± 16.3	195.3 ± 15.4	201.3 ± 14.1	**.014**	1336.3 ± 700.6	1413.9 ± 526.1	1238.7 ± 466.7	.949	1521.7 ± 716.8	1587.9 ± 467.9	1442.9 ± 470.5	.247
	Y	219.3 ± 5.4	216.2 ± 7.7	222.9 ± 3.9	.494	1301.4 ± 712.8	1455.6 ± 1099.9	1372.6 ± 759.4	.538	1547.4 ± 685.1	1690.7 ± 1115.2	1595.1 ± 764.4	.638
Protein (g)	O	5.1 ± 2.1	5.2 ± 1.9	5.4 ± 1.7	**.025**	51.7 ± 23.9	60.2 ± 28.3	49.9 ± 16.5	.603	56.7 ± 24.3	56.8 ± 21.3	55.5 ± 16.9	.739
	Y	5.9 ± 0.7	5.8 ± 0.9	6.1 ± 0.5	.807	92.9 ± 41.3	64.7 ± 42.9	64.9 ± 38.9	.334	70.1 ± 8.9	71.7 ± 9.8	71.6 ± 8.6	.317
Carbohydrate (g)	O	30.8 ± 12.1	32.5 ± 11.5	33.5 ± 10.6	**.013**	137.6 ± 93.9	142.2 ± 69.9	131.6 ± 65.6	.786	168.5 ± 96.5	173.1 ± 69.2	165.8 ± 65.1	.861
	Y	36.5 ± 4.1	35.9 ± 5.8	37.1 ± 3.1	.494	132.5 ± 73.6	167.1 ± 161.7	141.2 ± 102.9	.316	171.2 ± 70.4	205.4 ± 164.1	178.3 ± 103.7	.259
Fat (g)	O	4.7 ± 1.8	4.9 ± 1.7	5.1 ± 1.6	**.002**	56.1 ± 37.1	59.8 ± 25.6	57.3 ± 40.9	.675	60.7 ± 37.2	65.7 ± 26.9	62.4 ± 41.1	.638
	Y	5.5 ± 0.6	5.4 ± 0.9	5.6 ± 0.4	.441	56.9 ± 39.9	57.7 ± 46.5	60.1 ± 35.2	.767	63.1 ± 38.9	63.7 ± 46.8	66.2 ± 34.6	.705
Saturated fat (g)	O	0.4 ± 0.2	0.4 ± 0.2	0.4 ± 0.1	.058	21.1 ± 2.9	22.9 ± 2.6	18.9 ± 2.1	.353	21.5 ± 13.3	23.6 ± 11.9	19.4 ± 9.6	.369
	Y	0.5 ± 0.1	0.5 ± 0.8	0.5 ± 0.1	.441	21.1 ± 17.7	20.6 ± 26.2	21.5 ± 16.4	.308	21.7 ± 17.6	21.3 ± 26.1	21.6 ± 16.4	.350
Sugar (g)	O	3.4 ± 1.4	3.6 ± 1.3	3.7 ± 1.2	.161	71.6 ± 67.3	71.7 ± 39.7	59.7 ± 30.6	.711	74.9 ± 67.6	75.4 ± 39.5	63.6 ± 30.8	.705
	Y	4.1 ± 0.5	3.9 ± 0.7	4.1 ± 0.3	.494	60.2 ± 34.6	94.9 ± 124.2	57.9 ± 50.8	.711	64.4 ± 34.4	99.1 ± 124.2	61.6 ± 50.9	.705

Abbreviations: O, older Adults; SC, control soup; SSC, sumac added during cooking; SSE, sumac added at the end; Y, younger adults. Data are shown as mean ± SD and tested for normality using a Shapiro–Wilk's test, bold figures show statistically significant difference at *p* < .05 following repeated measures ANOVA and Friedman tests.

#### 3.4.2 Energy and nutritional profiles of ad libitum lunch, evening meal, and the all‐day food intake between participant groups

The macronutrient intake of ad libitum lunches, evening meals, and all‐day after the test sessions (SC, SSE, and SSC) between older adults and younger adults was analyzed (Table 5). Added CIBS revealed no statistical differences in nutrient intake between older adults and younger adults (*p* > .05) following ad libitum lunch. However, protein intake was higher during the evening meal following the SSC soup test (*p* = .003) with higher intake by younger adults than older adults. Furthermore, more intake of protein was observed after SSE (*p* = .006) and SSC (*p* = .004) sessions on all‐day food intake between the groups with greater consumption in younger adults.

**TABLE 5:** Mean differences in the ad libitum lunch, evening meal, and all‐day energy and macronutrient intake after the test sessions (SC, SSE, and SSC) in older adults (>65 years old) compared with younger adults (18–35 years old).

Nutrients	Meals	Test sessions between older and younger adults					
		SC	*p*‐value	SSE	*p*‐value	SSC	*p*‐value
Energy (kcal)	Ad libitum lunch	−33.9	.265	−20.9	.114	−21.6	.178
	Evening meal	34.9	.883	−41.7	.529	−133.9	.078
	All‐day	−25.7	.512	−102.8	.678	−152.2	.091
Protein (g)	Ad libitum lunch	−0.9	.174	−0.6	.211	−0.5	.398
	Evening meal	−11.2	.065	−4.5	.119	−14.9	**.003**
	All‐day	−13.4	.090	−5.9	**.006**	−16.1	**.004**
Carbohydrate (g)	Ad libitum lunch	−5.7	.265	−3.4	.114	−3.6	.718
	Evening meal	5.1	.640	−24.9	.698	−9.6	.758
	All‐day	−2.7	.289	−32.4	.989	−12.5	.841
Fat (g)	Ad libitum lunch	−0.8	.383	−0.5	.114	−0.5	.841
	Evening meal	−0.9	.883	2.1	.327	−2.8	.547
	All‐day	−2.3	.799	2	.478	−3.8	.512
Saturated Fat (g)	Ad libitum lunch	−0.07	.165	−0.03	.445	−0.03	.429
	Evening meal	0.02	.698	2.3	.063	−1.1	.968
	All‐day	0.13	.758	2.3	.068	−2.1	.968
Sugar (g)	Ad libitum lunch	−0.7	.102	−0.3	.114	−0.4	.102
	Evening meal	11.4	.758	−23.2	.758	1.8	.547
	All‐day	10.5	.925	−23.7	.779	1.7	.547

*Note*: Data are shown as difference between older adults and younger adults and tested for normality using a Shapiro–Wilk's test. T‐tests and Mann–Whitney tests were run to compare significant differences between both groups of adults within the added sumac samples. Bold figures show significant differences at *p* < .05. SC (Control soup), SSE (sumac added at the end), and SSC (sumac added during cooking).

## 4 DISCUSSION

The current study compared the food intake within and between older adults and younger adults and confirmed a reduction in food intake with aging, which has been reported previously (Giezenaar et al., 2016). The addition of spices such as sumac may compensate for the chemosensory impairment of older adult participants as the energy, carbohydrate, protein, and fat intake in the ad libitum lunch of these groups increased following consumption of soup with sumac, whereas the younger adults showed no changes. Sumac contains various volatile compounds that are released via the nasal route and trigger olfactory receptors (Bell et al., 2018; Farag et al., 2018). Additionally, the consumption of food flavored with herbs and spices stimulates the sensory receptors located in the mouth and enhances the food intake (Field & Duizer, 2016). Thus, it can be speculated that the addition of sumac to older adults' food increases its palatability, resulting in greater food consumption.

In the current study, a higher intake of protein was observed in older adults in an evening meal following the consumption of soup in SSE session. Therefore, it can be hypothesized that the SSE session had a greater impact on protein intake compared with SSC and SC. The importance of an adequate intake of protein for older adults' health and the prevention of malnutrition is highlighted in epidemiological studies (Baum et al., 2016). It has been reported that 35% of residents in care homes in Europe consume less protein than is recommended (0.7 g/kg of their body weight per day) (Tieland et al., 2012). The consumption of 1.0–1.2 g protein/kg of body weight per day by healthy older adults, increasing to 1.2–1.5 g/kg for malnourished older adults, has been suggested (Deutz et al., 2014). However, more investigation is required to substantiate the impact of added sumac on protein intake.

The findings of the current study demonstrated a marginally higher intake of energy, protein, carbohydrate, and fat in an ad libitum lunch among older adults following the addition of sumac in soup. In agreement, a positive influence of additive seasoning and sauce to older adults' food to enhance energy, protein, and fat intake was reported previously (Best & Appleton, 2011). The impact on the ad libitum lunch intake can possibly be explained by the presence of L‐glutamic acid in sumac, which *via* the activation of umami subunits and coupling with G‐protein stimulate appetite (Camilleri, 2015). On the other hand, the impact of polyphenol compounds in increasing glucagon‐like peptide‐1 hormone and decreasing ghrelin is reported (Boix‐Castejon et al., 2018) in addition to the rapid omission of polyphenols and their weak absorption into the body's circulation that are well documented (Hollman, 2014). Therefore, these paradoxical findings require further assessment to understand the bioavailability of the polyphenol compounds following consumption.

In the current study, the perception of intensity of brown color of sumac in soups was significantly different between the older and younger adults and within each group; however, no other differences were observed. One reason could be the small differences between doses (0%–1%) of sumac, so it was difficult to differentiate the brown color and flavor of the samples (Methven et al., 2016). The most common doses of herbs and spices intake generally ranged between 1 and 5% (Vazquez‐Fresno et al., 2019). Another reason could be the personal perception of taste and flavor preferences. Based on the variety of the chemical structure of aroma compounds, including acids, alcohol, and esters, various factors participate in the release of aroma from spices in foods. Moreover, multisensory interaction, for instance, olfactory, gustatory, and oral somatosensory, plays an important role in food flavor perception. This could be a reason why some participants found the samples more spicy, salty, or sweet. However, this finding requires more research on taste perception of sumac, which can be affected by genetics, cultures, and habitual intakes.

The addition of sumac in this study did not contribute to sour or lemony flavor in soup. This could occur due to the method of cooking and interaction of low‐salt vegetable stock ingredients in the soup (Opara & Chohan, 2014). The impact of viscosity on flavor recognition and the importance of organoleptic properties in food preferences have been reported previously (Bult et al., 2007). Volatile compounds in spices may interact with the food matrix via the saliva and nasopharynx pathway and, hence, flavor is released following olfactory activation. The interaction of sumac in the food matrix remains still unknown; however, the results of sensory evaluation could be explained by high levels of carbohydrate compounds in butternut squash which play a major role in sweetness and texture as a dominant perception over the sour and lemon flavor of small doses of sumac.

In this study, FBS and CIBS showed the highest levels of polyphenol content and antioxidant activity compared with all other samples justifying their use in human studies. The polyphenol content of water‐extracted samples in this study was similar to the results of Bashash et al. (2012), who found that the yield ranged from 0.811 to 2.45 mg GAE/g. However, Bashash et al. (2012) compared the phenolic compounds of brown sumac fruit, brown sumac powder, and red sumac powder extracted with four different solvents and demonstrated that water was the most efficient solvent, whereas in the current study 80% acetone and 80% ethanol were the most effective solvents for polyphenol and antioxidant activity, respectively. This may be due to solvent polarity, which has a major role in increasing solubility of phenolic compounds, depending on their structure (Zlotek et al., 2016). Acetone/water mixture is generally more effective in extracting more polar antioxidants from plant materials such as leaves, fruits, and vegetables, whereas ethanol/water mixture can solubilize a wide range of phenolic compounds (Alothman et al., 2009; Naczk & Shahidi, 2006). Considering the impact of diverse analytical procedures and ensuing previous studies, acetone/water (80/20, v/v) and ethanol/water (80/20, v/v) were chosen in the current study to obtain robust and comparable results. Water was used as another extractant to determine potential bioaccessibility of phenolic compounds and glutamic acid in human studies. Previous studies revealed that sumac contains nearly 200 different phenolic compounds including gallic acids (Bozan et al., 2003), hydrolyzable tannins, anthocyanins, and flavonoids (Ardalani et al., 2016). Therefore, the authors acknowledge the difficulty in extracting all the phenolic compounds using one solvent mixture and there may be variations in the amount and type of compounds depending on the solvent system used.

The results for L‐glutamic acid levels in the current study varied and were higher than earlier findings for Syrian and Chinese sumac (Kossah et al., 2010). The level of glutamic acid in Chinese sumac was significantly higher than in Syrian sumac suggesting that geographical location, environmental factors, and growing conditions of sumac may influence the levels of glutamic acid. Pedraza et al. (2007) used various methods including Megazyme assay for assessing L‐glutamic acid levels in foods and their results confirmed that the L‐glutamic acid was highly soluble and clearly detected without interference from other compounds in foods. Thus, it can be speculated that the Megazyme L‐glutamic acid kit used in the current study is an accurate, reliable, and uncomplicated technique to use. This study lays the groundwork for future research into sumac and the addition of this spice as a flavor enhancer in the food industry due to the presence of glutamic acid, which plays an important role in food palatability and acceptability (Ghawi et al., 2014). Moreover, the stimulation of secretion of immunoglobin A and saliva by glutamic acid has been reported to show an association between nutrition and improved immune status (Schiffman, 2000).

This is the first study seeking to ascertain the importance of the addition of sumac to enhance palatability and food intake among older adults in the short term. Future research could assess the impact of the addition of sumac to food over a longer period. Additional sensory studies need to be carried out in order to validate the acceptability and liking of higher doses of sumac in different foods. The authors acknowledge some limitations. The study was conducted among a particular demographic of participants, with most being educated and white population in Oxford, UK. The lack of diversity in the social backgrounds of participants in the current study diminishes the generalizability of its findings. The study was limited by the accessibility to commercial forms of brown sumac from other regions, similar to Iranian sumac. Moreover, it was not possible to obtain fresh sumac from areas other than the UK and Iran. Therefore, this would have caused a bias in selecting sumac with the highest concentrations of polyphenol compounds. Additionally, the specific phenolic compounds in sumac extracts were not identified in this study. Further studies are warranted to characterize these polyphenols using more robust methodologies such as high‐performance liquid chromatography or capillary electrophoresis (Naczk & Shahidi, 2006). Another limitation of this study was the self‐reported dietary intake, which may have resulted in inaccurate recording of consumed food. An extension of study intervention may improve the familiarization of sumac in older adults leading to higher food intake. Therefore, the influence of sumac consumption on food intake among older adults should be investigated using longer intervention periods.

## 5 CONCLUSIONS

The findings of this study highlighted that commercial brown sumac had higher polyphenol, antioxidant, and L‐ glutamic content among all commercial sumac samples chosen in this study. To the best of our knowledge, this study is the first attempt to compare the sensory evaluation of different doses of sumac by older and younger adults. The findings confirmed that the soup sample with commercial brown sumac (1%) was the sample most preferred by both groups of adults. Additionally, the increase in doses of sumac had no adverse effects on the acceptability and liking attributes of soup samples. It can be suggested that sumac, which contains L‐glutamic acid, is a promising natural additive, to replace salt, with the aim of increasing food intake in older adults and improving the taste and flavor of food. Hence, the outcome of this study is a promising approach in response to global health concerns over malnutrition in older adults, potentially contributing to reductions in healthcare costs.

## AUTHOR CONTRIBUTIONS

**Nasim Soleymani Majd:** Conceptualization (equal); data curation (lead); formal analysis (lead); funding acquisition (lead); investigation (equal); methodology (equal); project administration (lead); resources (equal); software (equal); validation (equal); visualization (equal); writing – original draft (lead); writing – review and editing (supporting). **Shelly Coe:** Conceptualization (equal); data curation (equal); methodology (equal); project administration (equal); supervision (equal); writing – review and editing (equal). **Helen Lightowler:** Conceptualization (lead); data curation (supporting); formal analysis (supporting); funding acquisition (lead); investigation (equal); methodology (equal); project administration (supporting); resources (lead); software (equal); supervision (equal); validation (equal); visualization (equal); writing – original draft (supporting); writing – review and editing (supporting). **Pariyarath Thondre:** Conceptualization (equal); data curation (equal); formal analysis (equal); funding acquisition (equal); investigation (equal); methodology (equal); project administration (equal); resources (equal); software (equal); supervision (lead); validation (equal); visualization (equal); writing – review and editing (lead).

## CONFLICT OF INTEREST STATEMENT

The authors declare that they do not have any conflict of interest.

## ETHICS STATEMENT

This study was approved by the Institutional Review Board of Oxford Brookes University.

## INFORMED CONSENT

Written informed consent was obtained from all study participants.

## Data Availability

The data that support the findings of this study are available from the corresponding author upon reasonable request.
